# Psychological Model of Phonosemantics

**DOI:** 10.1007/s10936-020-09701-y

**Published:** 2020-04-22

**Authors:** Pramod Kumar Agrawal

**Affiliations:** Universal Theory Research Centre, D-9, Lal Bahadur Nagar East, Jawahar Lal Nehru Marg, Jaipur, Rajasthan 302017 India

**Keywords:** Phonosemantics, Semantic values, Psychological interpretation of phonemes, Model of psychological mind, Sound symbolism, Semiotics

## Abstract

Phonosemantics is a school of thought which believes that each sound or phoneme carries a specific psychological impression allotted by nature. And these psychological impressions were used to evolve different languages. Work has been done on this ground, but there is still scope for further research into the subject. The paper presents a new hypothesis, explaining the psychological representations of all the important IPA alphabets. The paper proposes a model of psychological mind, on which all the basic phonemes are placed, enabling us to understand the basic relationship between psychological semantic values and their phonetic values. To prove the correctness of the allocation, the paper applies these semantic features to 245 words of different languages, along with some additional evidences. The paper resolves the confusion regarding the same name for different objects, different names for the same object, the question of arbitrariness, and other queries raised by modern linguists.

**Electronic supplementary material:**

The online version of this article (10.1007/s10936-020-09701-y) contains supplementary material, which is available to authorized users.

## Introduction

The theory propounded here suggests that each sound or phoneme in this universe has a specific meaning allotted by nature. The paper further presents the process how these meanings of individual phonemes are used to create words. It is explained that how the physical signals which may be visual, sound, smell or of any mode are reach of our intellectual mind through the route of biological and psychological levels; how the preconceived memories of different levels diversify the observed image. The paper resolves the objections of modern linguists, including ‘arbitrariness’ in word formation. The paper explains how we have multiple meanings for a single word, and multiple words for a same meaning.

## Contribution to Previous Knowledge

It is widely acknowledged that there is some connection between sound and meaning in a word (Woodroffe 2008; Science Daily 2006; Michael 1958). The modern world believes that a ‘combination’ of two or three phonemes should have a cognizable meaning (Dennis 2013; Koerner 1993; Essay 2017), but an individual phoneme cannot have any meaning. But the meanings of these ‘combinations’, which are called phonesthemes, are still undiscovered. Nuckoll (1999) said that *“the ‘oil’ in roil, boil, and oil be analysed as a phonestheme meaning ‘something liquid’”*, but we cannot justify the words—foil, coil, moil, soil, and toil. According to the research done by Dr. Magnus (2001), we have Upanishads (Flood 2003), Plato’s Cratylus Dialogue (360BCE), and a list of English phonesthemes (Wallis 1663). We have Velimir Khlebnikov (Weststeijn 1998), Dwight Bolinger (Bobbs 1950), and Hans Marchand (Anglia 1979), who tried to give specific meanings to different phonemes and phonesthemes. But no one ever could succeed to provide any ‘*convincing theory’* as Ohala (1994) demanded.

The paper contributes the subject in the shape of a newly developed universally applicable theory, which allocates specific psychological meanings to all individual phonemes in a tabulated form. It explains how these psychological meanings are used for pragmatic purposes. The meanings of phonesthemes can automatically be evolved with the help of the meanings of individual phonemes. The paper further presents the process how these meanings of individual phonemes are used to create words. The paper resolves the objections of modern linguists, including ‘arbitrariness’ in word formation in detail. The paper explains how we have multiple meanings for a single word, and multiple words for a same meaning.

## Process of Communication

### Psychological Meanings

Different sounds generate different feelings. Some sounds generate pleasure inside and some generate a fear inside. These feelings of pleasure and fear cannot be explained literally, but can be answered psychologically by crying or by laughing. In this way, we have a psychological interaction. All animal interactions and infant interactions come into this category. We know that, without a meaning, no interaction is possible. For the purpose of this paper, this ‘meaning’ is taken as ‘psychological meaning’. There is a difference between ‘feeling’ and ‘knowing’. ‘Feel’ is psychological and ‘know’ is intellectual. You cannot ‘know’ the thing unless you ‘feel’ it. The ‘know’ is an interpretation of ‘feel’. The ‘sense’ within ‘feel’ is called psychological meaning. Sometimes this ‘feel’ is called ‘sound symbol’; in other words, all ‘sound symbols’ have specific ‘psychological meanings’.

### Multiple Levels of Communication

**Fig. 1:**
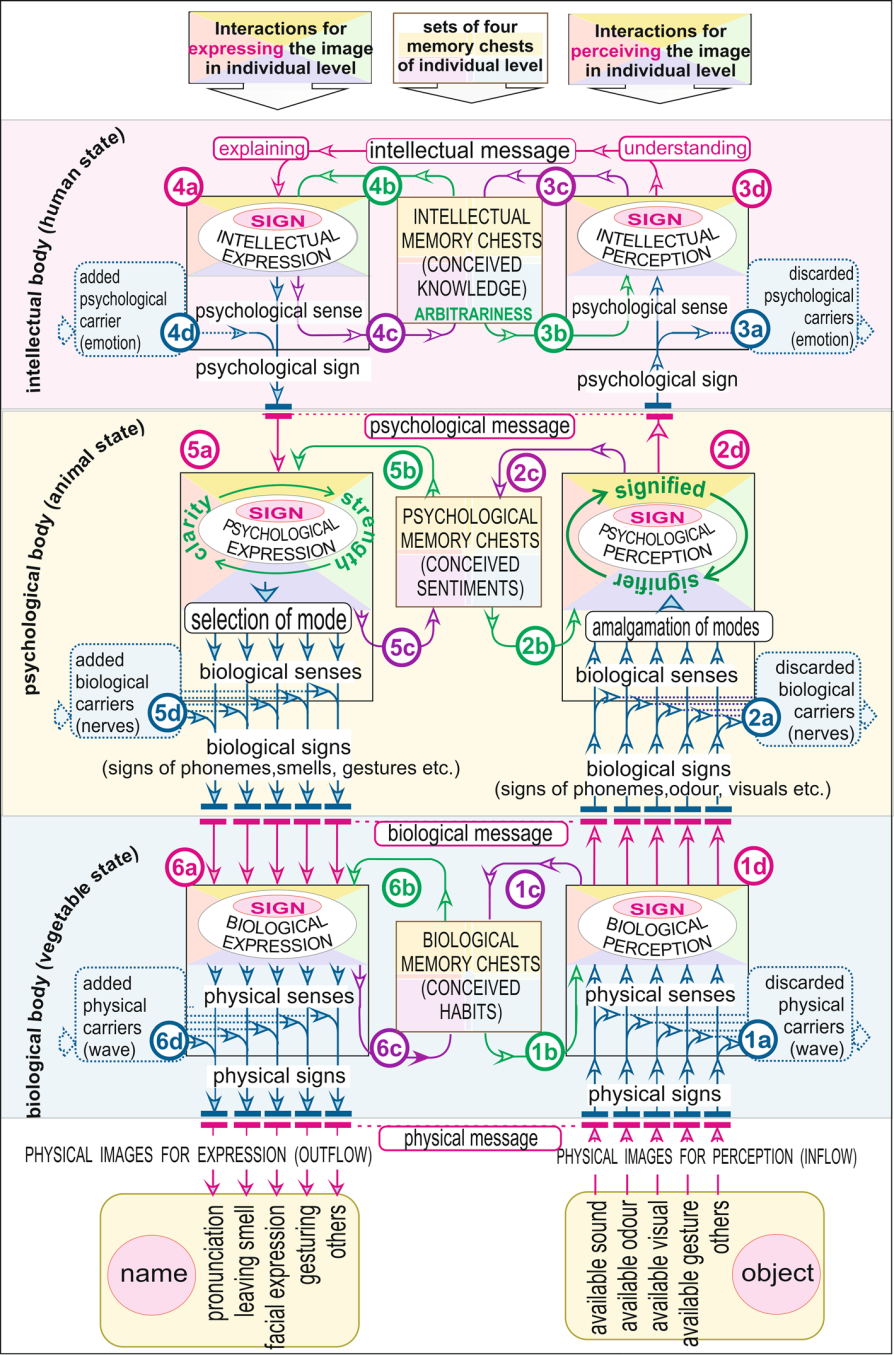
Impact of biological, psychological, and intellectual memories on perception and expression

A human perceives a sign using multiple levels of his entity (refer Fig. 1). The sign is neither the ‘thing’ (Langer 1951), nor ‘proxy’ for the object; these are just a pattern of feeling (Schechter 2008), which comes to us through biological, psychological and intellectual levels of our entity. This sign is made of two parts (Winfried 1990): ‘carrier (vehicle)’ and ‘sense’. Therefore, while perceiving a sign from physical object to intellectual mind, we have to go through following levels. Please refer Fig. 1, (1) At the biological level, the perceiver discards the ‘physical carrier (loudness)’ and perceives the ‘physical sense’ in the form of biological sign. (2) At the psychological level, the perceiver discards the ‘biological carrier (neuro-chemicals)’ and perceives the ‘biological sense’ in the form of psychological sign (feeling). (3) At the intellectual level, the perceiver discards the ‘psychological carrier (emotions)’ and perceives the ‘psychological sense’ in the form of intellectual sign or pragmatic meaning. In this way, the physical signal reaches the intellectual level step by step.

In the case of expression (outflow) of sign, all the process is reversed. At each level the sense finds an added carrier which converts the upper level sense to the lower level sign. The intellectual sense reaches the psychological level, biological level, and physical level in the form of ‘speaking’ (refer Fig. 1).

### Multiple Channels of Communication

Communication occurs when a signal carrying information travels from source to destination (Krauss 2002). There are multiple channels of communication. Animals including humans use different types of channel (https://study.com/academy/lesson/how-animals-communicate-chemical-visual-electrical-signals.html). They communicate through gesture, facial expression, smell, voice and other channels of communications (Introduction to Language 2015; Animal Communications 2002). The brain can perceive and interpret a stimulus with different channels of sensing (Sperling 1990). Having the same biological carriers, the same species can communicate psychologically without any problem (Palmer 2012). In all we have different channels for communication. We have smell language (Web of Life 2012), body language (Mondloch et al. 2013), and facial expression language (Dictionary of Scientific & Technical Terms 2003). All these languages are amalgamated (“Contribution to Previous Knowledge” section of Fig. 1) and transformed into psychological feelings. A ‘visual (ghost)’, a ‘facial expression (fearful)’, and a ‘sound (cry)’ can be converted into the same psychological feeling of ‘unprotected’. A ‘visual (funny)’, a ‘facial expression (smile)’, and a ‘sound (laugh)’ can be converted into the same psychological feeling of ‘pleasure’. It hardly matters from which channel a sign is coming for observation, they all amalgamated on the same psychological platform.

In the case of expression, the same psychological feeling can be conveyed with the help of any channel of communication. For example, the ‘sound’ of / n / and a ‘specific facial expression’ of ‘negation’, both signals can be used to convey the same psychological message of ‘negativity’. An animal can bark (sound language) or leave a specific smell (smell language) to express the same psychological message of ‘territorial clarification’ (Sillero-Zubiri and Macdonald 1998). When we try to express anything by speaking, facial expressions also come along. It is found that for a person, if one channel becomes ineffective or weak, other channels compensate its inactivity. Refer to Fig. 1.

### Process of Inflow (Perception)

In the case of an observation, the sign starts from an object and reaches our intellectual mind by passing through all levels of entity one by one. At each level the perception is influenced by preconceived memories (Mohammadi and Banirostam 2015).

In this way, while observing through biological level (Fig. 1), we get physical sense coming from two sources: (1) from physical [lower] entity situated outside (1a); (2) from preconceived biological memory [higher entity] (1b). After interaction, a biological sign is evolved. The biological sign so formed is sent to two distinct directions: (1) stored in the biological memory in the form of habit (1c); (2) offered for psychological perception (1d). The same process is repeated at all levels. And sense reaches to our intellectual mind. As per the Saussure’s theory (Fig. 1) (2a), the perceiver receives psychological impression by the evidence value of his senses (de Saussure 1974a, p. 66, 1983a, p. 66) in the form of association of the signifier and the signified (de Saussure 1974b, p. 67, 1983b, p. 67). It is clear that if there is no signifier, nothing will be signified (de Saussure 1974c, p. 102, 1983c, p. 101). The signifier is the perceiver, perceiving a sign within meaning, and the signified is a sign available having perceivability within some appearable form. Both are psychological.

### Process of Outflow (Expression)

The process of expression is just opposite to the perception. When we express something, the learned phenomena (preconceived expression data of all levels; 4b;5b;6b refer Fig. 1) are added to our outflowing signals, and the phenomena (conceived expression data at all the levels; 4c; 5c; 6c refer Fig. 1) are memorized at each level. Therefore, there can be a lot of difference between ‘to be expressed’ and ‘actually expressed’ (Smith and McIntyre 1984).

### Impact of Preconceived Memories on Perception and Expression

A visual perception cannot always be trusted (Zamora 2015; Gregory 1997; Illusions and Paradoxes), because the perceived image largely depends on the preconceived memories (Sperling 1990) (Refer Fig. 1). A psychoanalyst displays the same picture to different patients and finds an entirely different response from each one because each one has different preconceived memories. Everyone has relative truth; no one has the absolute truth (Lee 2003; Maser 2006). A human being can be observed as ‘black’, ‘handsome’, and ‘tall’. Some other person may observe him as ‘bald, ‘funny’, and ‘fat’. Both may be right, but both are different. There can be following preconceived conditions at all the levels of the entity (Agrawal 2016, 2018).

1. Biological needs (geographical situation, food, activity, sex, etc.)
2. Biological availability (biological codes (DNA), organs for perception and expression, environmental conditions, habit, etc.).
3. Psychological needs (protection, fulfilment of desire, companion, ego satisfaction, etc.).
4. Psychological availability (ego, courage, faith, character, social values, learned emotions, etc.).
5. Intellectual needs (curiosity, purpose, etc.).
6. Intellectual availability (education, notions, intelligence, etc.).

As far as the animals of the same species (including human beings) are concerned, the first two factors are more or less the same; the third and the fourth may differ; and the fifth and the sixth are not available. Having the same biological built up, while expressing, they produce resembling psychological signs. Hence the psychological communication becomes possible.

As far as the humans are concerned, our biological set ups are the same, hence we have the ability to talk psychologically like other animals. But our psychological set ups are not the same, hence we do not have the ability to talk intellectually. To get rid of this problem, we adopt the psychological impressions in specified directions so that maximum people of the society can correlate the psychological impressions and adopted meanings.

### Use of Arbitrariness

Theoretically, all persons have different preconceived memories at all the three levels: the biological, the psychological preconception, and the intellectual level. Each psychological sign may convey multiple intellectual meanings. Different persons having different preconceived memories can select different pragmatic image. For example, the psychological feeling behind the sound / k / can be arbitrarily defined as consciousness’, ‘alertness’, ‘precaution’, ‘analysing’, ‘awareness’ etc. Every different person may select any pragmatic image according to his purpose. This selection is called ‘arbitrariness’.

## Removal of Doubts

### Natural and Arbitrary Symbolism

Chandler (1994) explains many things what Saussure had said about the ‘arbitrariness’. We do not find any difference between Saussure’s thoughts and the hypothesis taken in the paper. Please refer pera 3.6 and pera 3.7.

### Naming an Object

Animals communicate psychologically in pure natural ways (Robert 1999). Their communication purely depends on psychological feelings and sentiments like fear, desire and sex. As regards humans, they have one additional level, which is called the intellectual level and which tries to understand these psychological signals in a logical and pragmatic manner. It is suggested that the same psychological sign can be used for multiple pragmatic meanings. Out of these multiple pragmatic meanings, every individual human can arbitrarily select any meaning which suits his purpose. For example, when you see something which is like ‘liquid’, ‘transparent’, ‘invisible’, ‘life’, hydrogen dioxide, cleaning material. If your purpose is towards ‘visibility’, you will select ‘invisible’ out of them. Your psychological definition will be “involvement in the tendency of invisible expression”. In other words, its phonetic value will “/ r / involvement in the / tǝ / tendency of / wa / invisible expression”, that is / w a t ǝ r /. And the intellectual sign / w a t ǝ r / will be evolved. The so evolved intellectual sign is not a sound pattern. Without knowing any language, you can keep it in your mind. This intellectual impression is called ‘knowledge’. When this knowledge is offered for expression, it passes through the intellectual platform (“Removal of Doubts” section of Fig. 1), and reaches the psychological platform (“Model of Phonosemantics” section of Fig. 1). Here the mode of expression is to be selected. If the selected mode is sound, the platform provides corresponding phonetic features to the expression. When this ‘phonetic feature’ is pronounced physically, it is called ‘name’. This name is memorized in the biological memory. Now we have learned the name / watǝr / for the liquid.

That is why it seems to be difficult to convert physical gestures into psychological feelings because we have already affixed different words for our pragmatic purposes. But the languages were developed around 60,000 years back (Bryant 2010; Nowak 2001). The fact shows that this was the period when humans were converting themselves from animals to humans. We might be very efficient in converting physical gestures into psychological feelings just like other animals. Even today we can easily convert a facial expression or body gesture into a psychological feeling.

*Explanation 1*. According to linguists, *“the name ‘dog’ is arbitrary and it has nothing to do with the ‘concept of dog’, except that a speech community has agreed that this name represents that conceptual meaning”*. The paper suggests that the ‘concept’ has no real value, but the phrase “*speech community has agreed”* has importance in it. If the persons of the speech community belong to the same geographical situations, environmental conditions, and social values, they will all find more or less the same psychological image and will agree on more or less the same name. The people of different countries may get different psychological images and may agree on different names.

*2*. In a discussion Socrates says, “*Well, if anyone could express the essence of each thing in letters and syllables, would he not express the nature of each thing?”* Margaret Magnus (2001) showed her disagreement. We feel a little misunderstanding between the two. When Socrates talks about ‘nature’, he talks about the ‘observing nature (signified)’, not the ‘existing nature (object itself)’. The ‘existing nature’ of chair may be the same for different cultures, but the ‘observing nature (signified psychological image)’ may not be the same. We feel a little mistake on the part of Socrates, that he used the word ‘nature’, in place of ‘part nature’ because a person observes only partial and familiar parameters of the psychological image (Spearling 1990).

### Recognizing Unknown Object by Its Name

It is wrong to presume that we can recognize any unknown object by a known name using phonosemantics. Phonosemantics does not connect object and phonemes; a bridge of psychology connects them. An object has infinite number of parameters; out of which two or three parameters are involved in naming it. These parameters can also be used for different objects. Hence the name does not represent the object itself; it represents the perception in the mind which may differ from person to person.

### Why There are Multiple Languages?

It is generally argued that if we have a specific meaning for all sounds, we should have a single language in the universe. Yes, there is a single universal language containing trillions of words. Different groups of persons, living in different environmental situations have selected different sets of words (made of some million) and determine particular set as their own individual language. In other words, we have a single universal language, and all the prevailing languages are a small or tiny part of that universal language.

### Proof of the Theory and Check of Falsifiability

The paper provides nature allotted psychological interpretations or semantic features of almost all phonemes. Our statement is that “every phonetic feature has a specific psychological semantic feature allotted by nature, and the root psychological meaning of a word can be understood with the help of these allotments”. This statement has certain conditions. (1) we must have an accurate IPA conversion of the word, (2) the word must have been naturally evolved, (3) we must know all possible pragmatic meanings of the word because difference in arbitrary selection will lead us to a different pragmatic meaning. To test the falsifiability, we can take a word and try to convert its phonetic features into psychological semantic features. By using the above theory, we can reach multiple but limited number of meanings. Now if you assign some incorrect semantic features to the phonetic features, you will not able to explain the pragmatic meaning of the same. For example, the theory suggests that phoneme / n / represents emptiness, eagerness to acquire, and capability to acquire. If you replace these meanings with consciousness, intensity or submission, you will not able to explain any word containing the sound / n /. The author has checked the semantic features for around 3200 words of different languages: English, French, German, Hindi. Please refer ‘Phonosemantic dictionary” (Agrawal 2018). The paper provides 245 words of English, French and German.

### Some Important Clarifications

We have used many words like clarity, strength, appearance, identity, flow, etc. All these words are used as segments of psychological image. For example, (1) ‘appearance’ can be defined as an unidentifiable gesture to be perceived (signified); (2) ‘identity’ can be defined as unappearable formulation (properties) of the image (signifier); (3) ‘clarity’ denotes logical diversity of the image; (4) ‘strength’ is the intensified stability in the image. Composition of (1) and (2) creates ‘perception’, and composition of (3) and (4) creates ‘expression’. The ‘perceived expression’ or ‘expressed perception’ both create the psychological image.

Psychological image itself is a big question mark. It is not what we visualize. Just after the formation of the psychological image, you can feel it, but you cannot recognize it. There is a large number of feelings one over another. You cannot describe any feeling. Your intellectual mind selects arbitrarily some of the purposeful feelings, and you reach a position to describe that feeling. A psychological image is just like dreaming. You dream all the night, but cannot understand what you dream, because all are psychological images. If your ‘signifier’ is partly open, and you are able to correlate some of them with the real life, you can remember that part in the morning.

## Model of Phonosemantics

### Structure of Individual Entity

**Fig. 2:**
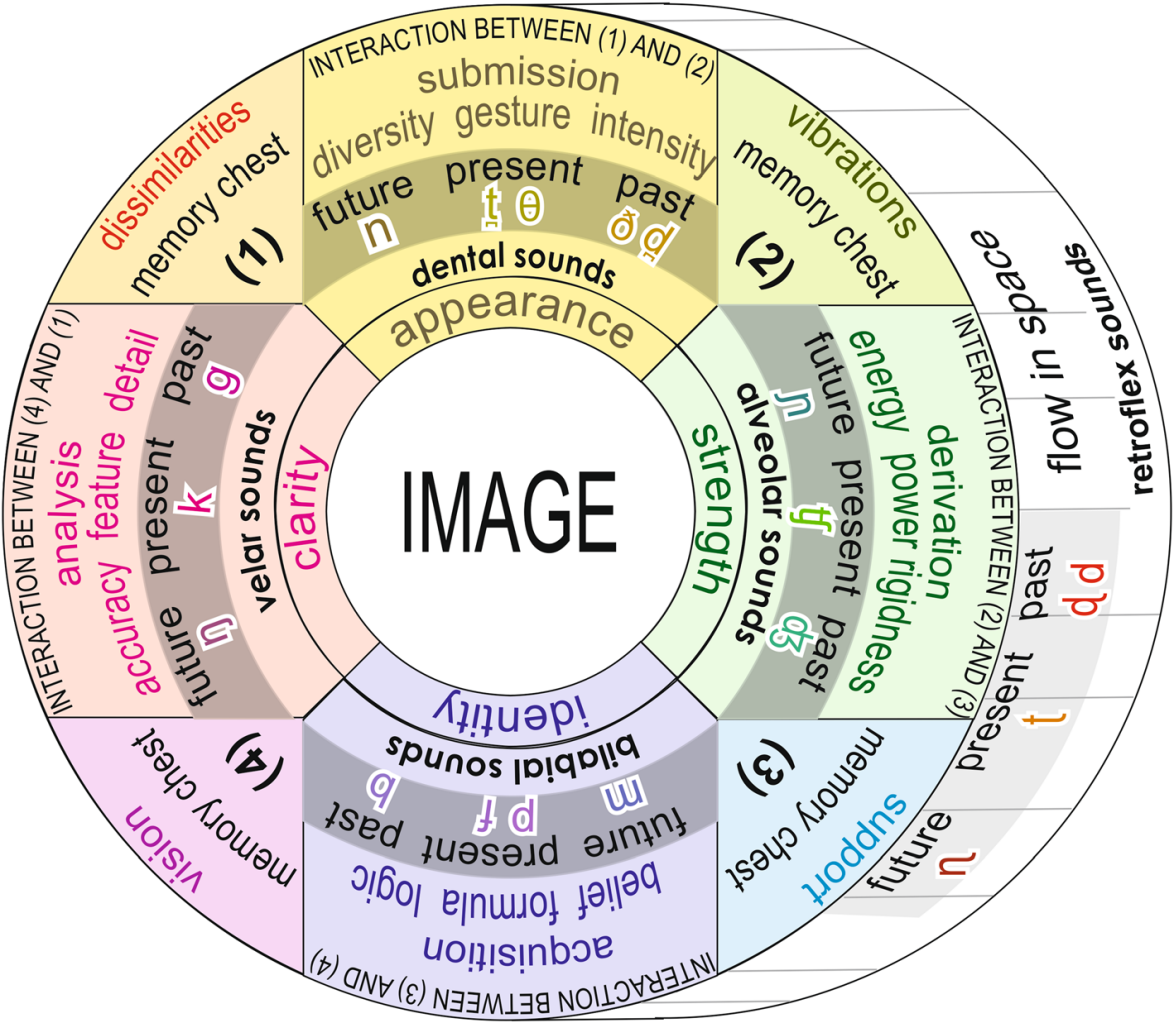
Palcement of consonants on the psychological platform

A human being is made of a number of levels: physical, biological, psychological, and intellectual level. Every level has its own entity. The theory suggests that an entity is made of (1) an interaction platform, and (2) five (four plus one) memory chests (Ojha 1987). Both of them represent consonants as well as vowels. In the case of matter (consonants), It is suggested that each unit of sign can be disintegrated in four parts in such a way so that when they are kept apart in the four different chests, they remain ‘inexpressible’ or ‘dormant’ (in the form of memory). But if all parts reach the interaction platform and join hands as one unit, they are expressed. The four chests are structured in such a way that each one receives, stores, and provides a different but specific aspect of a sign. (refer Fig. 2).

*Explanation*

1. Here the ‘chests’ and the ‘platform’ are only philosophical terms, and do not have any physical and situational implications in the brain.
2. When we divide a memory, we do not divide the memories. That is the division is within one memory only.
3. The four chests have the capability to receive, store and provide specific inexpressible data from/to interaction platform.

### Model of Consonants

Please refer Fig. 2 where it is explained that the structural model of consonants provides us five (four plus one) memory chests: (1) chest of inexpressible dissimilarities, (2) chest of inexpressible vibrations, (3) chest of inexpressible support, (4) chest of inexpressible vision, and (5) chest of inexpressible stimulation (Shastri 1987). The data stored in the four separated chests, are called dormant psychological memory and cannot be expressed with any phoneme. The moment some inflow data add to it, stimulation starts. It stimulates interaction between every two successive chests. And we get five (four plus one) different expressible constituents (appearance, identity, clarity, strength and flow) of psychological perception (refer Fig. 2). These five expressible constituents allot five different groups of sounds: dental, bilabial, velar, post alveolar, and fricative sounds respectively. These five groups are formed in such a way so that all the available psychological impressions can be explained within these groups. In this way the psychological ingredients (semantic features) are converted into five different groups of vocal sounds (phonetic features). For the purpose of symbolic representation, we have used IPA symbols which are taken from Handbook of the International Phonetic Association (2007), and from Aslam and Kak (2009).

The five basic constituents of any psychological image are as follows:

1. Interaction between psychological dissimilarities and vibrations evolves psychological constituent of submitted appearance, represented by the group of dental sounds / t̪ θ ð d̪ n /
2. Interaction between psychological support and vision evolves psychological constituent of acquired identity, represented by the group of bilabial sounds / p f b m /
3. Interaction between psychological vision and dissimilarities evolves psychological constituent of analysed clarity, represented by the group of velar sounds / k g ŋ /.
4. Interaction between psychological vibrations and support evolves psychological constituent of derived strength, represented by the group of post alveolar sounds / ʧ ʤ ɲ /.
5. Interaction between psychological space and flow of the above four evolves psychological constituent of occupied flow, represented by the group of retroflex sounds / t ɖ d ɳ /.

These are basic sounds. All other sounds are combinations of different sounds representing different psychological expressions in accordance with the sounds used therein.

### Model of Vowels

**Fig. 3:**
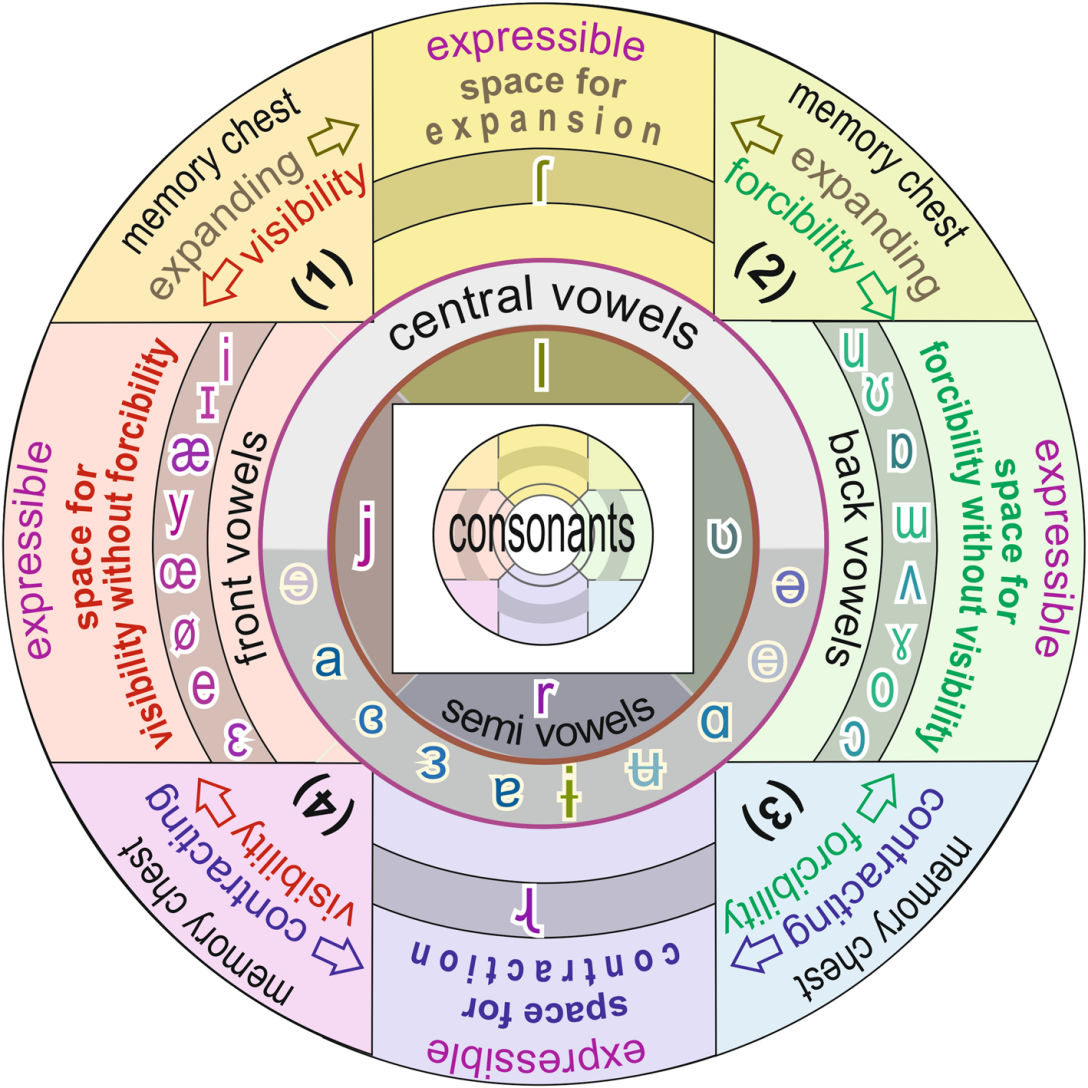
Distribution of consonants at the psychological platform

Vowels provide the different types of ‘fields’ to the consonants. We have five chests representing inexpressible ‘expansion visibility’, ‘expansion force’, ‘shrinkage visibility’, ‘shrinkage force’, and ‘empty space’. Due to interaction between every two successive chests, they form five different types of expressible psychological fields. Each type of field can support all types of consonants. Each group of fields represents the specific group of vowel-sounds Fig. 3.

Fields in the psychological perception are created as follows:

1. Interaction between ‘expansion visibility’ and ‘shrinkage visibility’ evolves ‘visibility without force’, represented by the group of front vowels / ɪ i e ɛ æ /.
2. Interaction between ‘expansion force’ and ‘shrinkage force’ evolves ‘force without visibility’, represented by the group of back vowels / ʊ u ɔ o ɒ /.
3. Interaction between ‘expansion force’ and ‘expansion visibility’ evolves ‘repulsion’ or ‘expansion’, represented by vowel sound / Ɩ /.
4. Interaction between ‘shrinkage force’ and ‘shrinkage visibility’ evolves ‘attraction’ and ‘concentration’, represented by vowel sound / ɻ /.
5. Interaction between the emptiness for the existence of all vowels, represented by the group of central vowels / ǝ ɜ /.

### Time Segment

Please refer Fig. 2. All our activities start from the future, move through the present and end in the past. All nasal consonants / n m ŋ ɲ ɳ / represent the future. All unvoiced consonants / t̪ p k ʧ t / represent the present. All voiced consonants / d̪ b g ʤ d / represent the past. In this way, all the five groups cover all the three segments of time.

### Positivity and Negativity

Every voiced and unvoiced sound can be divided into two parts. The un-aspirated sounds denote positivity and aspirated sounds denote negativity. For example, / k / denotes ‘analysing with open consciousness’, and / k^h^ / denotes ‘analysing with closed consciousness’. Here negativity can also be defined as ‘place provided for positivity’.

## Semantic Representations of IPA Sounds

We have divided the IPA script in such a way that each group of phonemes represent a specific part of psychological perception. Please note that all the semantic features and the corresponding phonetic features are provided in the same colour.

### Group of Submitting Appearance (Dental Sounds)

Interaction between ‘dissimilarities (chest 1)’ and ‘vibrations (chest 2)’ creates psychological appearances (diversity; gesture; intensity). Appearance can be converted into an image only when the ‘identity’ acquires it within its limitations. (Please refer Fig. 2)

1. / n / - (nasal sound; the future; no submission) unavailable appearance; emptiness; fullness of identity; eager to acquire; acquisition capability; act of acquiring; negation; near; small; few.
2. / t̪ / - (unvoiced sound; the present; unestablished offering)—towards offering/submitting the appearance (diversity; gesture; intensity) with unestablished (formless; shapeless; un-composed) display (sign; signal) in the present.
3. / θ / - (unvoiced sound; the present; established offering)—towards offering/submitting the appearance (diversity; gesture; intensity) with established (formed; shaped; composed) display (sign; signal) in the present; decided.
4. / d̪ / / ð / - (voiced sound; the past; unestablished submission)—offered/submitted appearance (diversity; gesture; intensity) with unestablished (formless; shapeless; un-composed) submission (sign; signal); already offered.

### Group of Acquiring Identity (Bilabial Sounds)

Interaction between ‘vision (chest 4)’ and ‘support (chest 3)’ creates psychological identity (logic; formulation; belief). Identity can be converted into an image only when there is ‘appearance’ to be identified. (Please refer Fig. 2)

1. / m / - (nasal sound; the future; unacquired availability)—unavailable identity; eagerness to be acquired; submission capability; submitted availability; having; available offerings, surrender; substance; disapproved; submissively.
2. / p / - (unvoiced sound; the present; approval with condition)—towards acquiring (adopting; approving; allowing) the identity (logic; formulation; belief) with conditions (protection; bond; support; check; restriction); approval.
3. / f / - (unvoiced sound; the present; approval without condition)—towards acquiring (adopting; approving; allowing) the identity (logic; formulation; belief) with no-condition (unprotected; without bond; unsupported; no-security; no-check; no-restriction; danger; fear; free); illogical acquisition; fast; sudden.
4. / b / - (voiced sound; the past; approved with condition)—acquired (adopted; approved; allowed) the identity (logic; belief; formulation) with condition (protection; bond; support; security; check; restriction) in the past; compulsion; bonding; principle; limitation; nature; helplessness, biased; confined; controlled; honourable; immovable.
5. / v / - (voiced sound; the past; acquired inside)—invisible expression/nature; invisible acceptance; hidden capability; kept expression; faith expression; soul; alive expression; storing; accommodation.

### Group of Analysing Clarity (Velar Sounds)

Interaction between ‘vision (chest 4)’ and ‘dissimilarities (chest 1)’ creates psychological clarity (accuracy; feature; detail). This is consciousness of our mind governed by curiosity about the subject. (Please refer Fig. 2)

1. / ŋ / - (nasal sound; the future; need of clarity)—unavailable clarity (accuracy; feature; details); eagerness for analysis; curiosity; capability for analysis; confusing; continuous; emotionally strong; fullness of strength; lively.
2. / k / - (unvoiced sound; the present; analysis with open consciousness)—towards analysing (clarifying; explaining) the clarity (accuracy; feature; details) with open consciousness; yet to be analysed; disintegrating; question mark; attentiveness; precautious; alertness; hesitating; digesting (biologically); getting fear (towards over precautious).
3. / g / - (voiced sound; the past; clarified open consciousness)—analysed (manifest; clarified; noticeable; organized) clarity (details; feature; accuracy;) with open consciousness; clarified non-strength (insecure; threat); object; defined; result; decision; clarified non-pleasure.
4. / ϰ / - expressible consciousness.

### Group of Deriving Strength (Post Alveolar Sounds)

Interaction between ‘support (chest 3)’ and ‘vibrations (chest 2)’ creates psychological strength (rigidness; power; energy) (Gurutu 1981). This is the liveliness of our mind governed by courage towards the subject.

1. / ɲ / - (nasal sound; the future; need of liveliness)—unavailable strength (energy; power; firmness); fullness of clarity; eagerness to derive strength; capability to derive strength.
2. / ʧ / - (unvoiced sound; the present; deriving liveliness)—towards deriving (achieving; attaining; collecting; invoking) strength (energy; power; firmness) with streamlined liveliness; courage; vibrating energy; wave energy; charge; towards deriving illusion (inattentive); pleasure; picking; provider; repetition.
3. / ʤ / - (voiced sound; the past; derived liveliness)—derived strength (energy; power; firmness) with streamlined liveliness; consistent energy; derived lack of clarity (fantasy); aliveness (psychological strength); pleasure; unclear derivation.

### Group of Occupying Flow of Time (Retroflex Sounds)

Interaction between ‘dissimilarities (chest 1)’ + ‘vibrations (chest 2)’ + ‘support (chest 3)’ + ‘vision (chest 4)’ with ‘stimulation (chest 5) creates psychological occupation (space, flow, time). This is the space in our mind which allows the activation and occupation. (Please refer Fig. 3)

1. / ɳ / - (nasal sound; the future; need of flow)—unavailable flow; fullness of space in existent; eagerness to occupy; execution capability; opportunity; punctual; spatial; eagerness for activation; extensive.
2. / t / - (unvoiced sound; the present; activation in the present)—towards occupying space with free fluency (inflow; outflow; self-flow) of the present; towards occupying; activating; doing; presence; Inclined; tendency; status; occupiable; achieving time; activated [/ t / is alveolar (nearly dental), hence a small amount of ‘offering’ is to be added while imagining its semantic value]; flow
3. / d / - (voiced sound; the past; occupied in the past)—already occupied space with free fluency (inflow; outflow; self-flow) in the past; occupied; activated; had; death; old; already happened; already existing; flown; available from past; status; state; done; achieved time; inclined; formed image; [/ d / is alveolar (nearly dental), hence a small amount of ‘offering’ is to be added].

### Composite Sounds (Other IPA Sounds)

Apart from the above basic sounds, we have a large number of composite sounds. In case of fricative sounds a trace of ‘physical expression’ is to be added. We are taking eight composite sounds here.

1. / z / - lively expression; expressible aliveness; continuous aliveness.
2. / ʒ / - expressible derived strength (energy; power; firmness); experiencing aliveness; energetic expression.
3. / w / - invisible expression; unidentified expression.
4. / s / - expressible; application of physical clarity; sense of physical expression; clear/visible expression; knowing phenomena; outside expression.
5. / ʃ / - application of physical strength; time of physical energetic experience; physical lively/believable expression; physical execution; learned phenomena; departed; lively past experience.
6. / s̪ / - application of physical flow; space of physical desire; occupiable/activated physical desired; pervaded physical expression; pervasion; sex phenomena; penetrant; interaction; permeation; desire; passion; will power.
7. / h / - application of placing into physical/non-existent (asata); availability in physical; death; removal from existence; elimination; hurt; outflow; towards non-life; leaving; loss; physical movement; entrance in physical placed; big; material body; relatively physical; inflow; gain.

### Semivowels

According to phonosemantics, vowels are a representation of ‘fields’ and hence semivowels are the ‘existence of different fields’. Refer Fig. 3

1. / j / = / ɪ + ǝ / - displaying acceptance; observable existence; visible without force existence; evident; direct.
2. / l / = / Ɩ + ǝ / - fullness of available expansion of appearance (emotion; motion; emission; spread; dispersion); lack of concentration; ignoring; expandable; emission; non-intellectual; apparent availability.
3. / ʋ / = / ʊ + ǝ / - invisible (hidden; inflow; force) existence; acceptable existence.
4. / r / = / ɻ + ǝ / - fullness of acquired concentration (involvement; logic; sensation; centralization; collection); intelligence; fineness; lack of expansion; monistic; by; dark (opposite to / l /); attraction (in Sindhi); identified.

### Group of Central Vowels (Self-existing) Refer Fig. 3

1. / ǝ / - existence; existence without existent [in English, ‘existence’ is more important, in Hindi ‘without existent’ is more important].
2. / ɜ / - availability of existent; available existent.
3. / ɜːr / - influenced existent.
4. / ɐ / - having; doer; being; involved entity.

### Group of Front Vowels (Visibility without Force) Refer Fig. 3

1. / ɪ / - visibly existing existent; visible (towards; in view; without force) existent; noticeable existent; evident; manifest.
2. / i / - exposing existent; outflow; executing; out exposing.
3. / e / - indicated/indicative/specific existent; straight; existence of visible existent; display; significative existent.
4. / ɛ / - visibly available existent; indicating existent.
5. / æ / - visibility of existent; outflow of existent.
6. / y / - visible acceptance; affirmation.
7. / a / - entity (with a little impression of outflow); existence with existent; executing existent; placement; doer; ness.

### Group of Back Vowels (Force without Visibility) Refer Fig. 3

1. / ʊ / - inside (hidden; inflow; indirect; invisible) existent.
2. / u / - accepting existent inside (inflow; internal; inner).
3. / o / - indirect/hidden/invisible/acceptable indicated existent; towards existent; non-indicative; direction; for.
4. / ɔ / - acceptable (invisible; forcible; inflow-able) availability of existent; invisible avail-ability of existent; perceivable availability.
5. / ɒ / - accept (hidden; inflow; inside; invisible) ability of existent.
6. / ʌ / - disclosure (growing; evolved; revealed; raised) of existent.
7. / ɑ / - entity (little impression of inflow); self-flow; by the entity; through the entity.

### Group of Devanagari Extra Vowels

1. / ɻ / - self concentrating existent; attracting existent; shrinking existent; centralization.
2. / Ɩ / - repulsive existent; self-expandable existent; inflated existent; dispersal; departing.

### Other Sounds

1. / ç / - expressible lively sensational; precision.
2. / ɑ̃ / - nasal sounds represent emptiness; desire; required; continuous.
3. / œ / - affirmation

## Experiments and Evidences

### Check for Falsifiability

We have already explained the falsifiability in chapter 4.5.

### Application of Semantic Values on Different Words

**Fig. 4:**
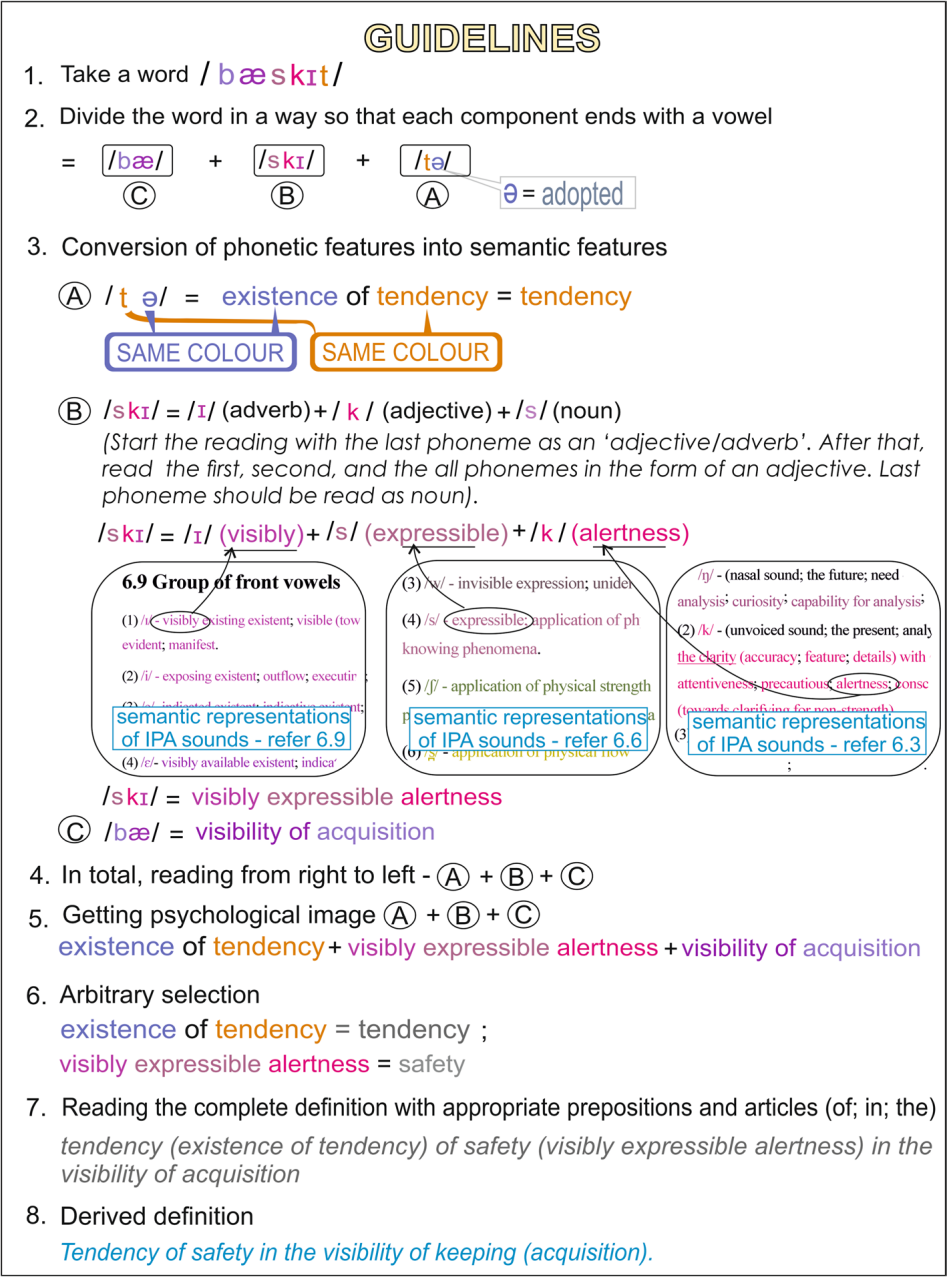
Guideline

We have provided semantic features of all the basic phonemes. We have explained (refer Fig. 4) the process by which the root pragmatic meaning of a word can be discovered. The Fig. 4 shows how the word ‘basket’ can be converted to its root meaning with the help of phonosemantics. The following appendixes provide 245 words, where the semantic values are taken according to the chapter 6 of this paper and the root meanings of the words are explained. Supplementary material: Appendix A—list of phonesthemes / fl /; Supplementary material: Appendix B—list of phonesthemes / pr /; Supplementary material: Appendix C—list of phonesthemes / gl /; Supplementary material: Appendix D—list of English words; Supplementary material: Appendix E—list of French words; Supplementary material: Appendix F—list of German words; Supplementary material: Appendix G—list of word ‘father’ in different languages.

### Animal Communication

It is a well-known fact that animals of the same species living in different countries can communicate without knowing any language (Palmer 2012). This can only be possible only when nature has provided them with the same phono-semantic conversion. Although their communications are just psychological, it verifies that it is nature who has allotted the semantic features, not the human.

### Animal Sounds

As we know that animals communicate purely in a psychological way. Most of their sounds relate to the safeguard of territory, threatening others, and their own introduction. We know that the meanings of the animal sounds are based on our own imagination, and cannot be used as the main evidence. But these can be used as corroborating evidences. We can take some animal sounds (Wikipedia 2018) as given in Supplementary material: Appendix H.

### Physical Sounds

We know that the sound was available even before the evolution of vegetation. Sound would have been made along with their meanings. Hence physical objects themselves say something while producing sound. Please refer Supplementary material: Appendix I. All the physical sounds are taken from Onomatopoeia Dictionary (n.d.).

### Dhatus and Phonesthemes

According to Indian mythology, dhātu can be defined as the smallest part of a word, representing the basic explainable meaning. Pāṇini (400BC) of India discovered 4000 dhātus. It can be confirmed that the meaning discovered by Pāṇini has been just equivalent to the present meaning of different world languages. For example, Pāṇini gave us ‘pā’ dhātu, with the meaning ‘to protect’. We have ‘palm’ (expanded submission for protection/approval), and ‘parcel’ (expanded availability involving the expression of protection; the things are protected inside) using the same meaning of ‘pā’. In the book ‘Meaningfulness of Sounds’, the author himself explained 100 dhātus on the basis of the present theory.

### An Individual Phoneme has the Same Meaning all Over the World

The basic meaning of sound / n / is negation (not having anything; emptiness). We have taken the word ‘negation’ of 57 countries where the conversion of negation resembles as follows:

Arbic – nafy, Azerbaijani – inkar, Bosnian – negacija, Catalan – negació, Cebuano – negation, Crosican – negazione, Croation – negacija, Czech – negace, Danish – negation, Dutch – ontkenning, Esperanto – negado, Filipino – negasyon, Finnish – negaatio, French – negation, Frisian – negaasje, Galician – negación, German – negation, Gujarati – nakāra, Haitian Creole – negasyon, Hausa – negation, Hawaiian – negation, Hindi – nakaar, Hmong – negation, Icelandic – neitun, Igbo – negation, Italian – negazione, Javanese – negasi, Kannada – Nirākaraṇe, Kurdish (Kurmanji) – neyînî, Lao – negation, Latin – negation, Latvian – negācija, Lithuanian – negacija, Uxembourgish – negatioun, Macedonian – negacija, Malagasy – negation, Malayalam – niṣēdhikkuka, Maltese – negazzjoni, Marathi – nakāra, Myanmar (Burmese) – negation, Nepali – namana, Norwegian – negasjon, Polish – negacja, Portuguese – negação, Punjabi – nakārātamaka, Romanian – negare, Scots Gaelic – neadachadh, Serbian – negacija, Sinhala – niṣēdhanaya kirīma, Slovak – negácia, Slovenian – negacija, Spanish – negación, Sundanese – negation, Swedish – negation, Uzbek – inkor qilish, Welsh – negation, Yiddish – negation.

## Results and Discussion

The definitions of the words derived with the semantic values seem to be quite satisfactory. This proves that the semantic features allotted to the phonetic features are appropriate up to high level of correctness. While going through the above experiment, the reader may be unhappy because of the following reasons:

1. The output of the words may seem to be questionable and obscure. This is because the psychological explanations touch the roots, without imposing any literate definition. There may be a wide difference between the root meaning and the present meaning. For example, originally ‘budget’ was a leather bag, where the money was kept safely, but today the ‘budget’ means how precisely we should spend the money. Although the root meanings in both the cases are the same, it seems questionable.
2. Sometimes it is argued that the explanations provided are far from accuracy. The author acknowledges this. It is because vocalization is also a type of gesture. It is just like the facial expression and body gesture. We can correlate a smiling face with psychological representation of pleasure. But pleasure can be of many types, which cannot be discriminated. The observer perceives the ‘smile’ within his own understanding. In other words, all the observers have different directions of ‘accuracy’; the definitions cannot satisfy all.
3. Is it a conclusive evidence? We have millions of words, out of which 245 words may not give us any conclusive proof, but it ensures correctness up to a high level of satisfaction. The author has published a phonosemantic dictionary (Agrawal 2018) which explains about 3200 words of different world languages. It is found that about 80% words can be explained by the theory suggested in the paper. However, we have artificial words, incorrect IPA conversion, and other factors, which decrease the percentage.
4. We have used some key words like ‘clarity’, ‘strength’. The reader has to add a word ‘psychological’ before these words. It is not ‘clarity’, but it is a ‘feel of psychological clarity’. Perception itself is not an image, but it is a ‘feel of psychological image’. Out of multiple felt images, we select a purposeful image, which is called visualized image. We just try to correlate the semantic gestures and the vocal gestures. Both are gestures only.

## Conclusion

The paper presents a new observational discovery in the field of natural language using empirical verifiability. The paper establishes that each sound (vocal gesture) has a natural specific psychological interpretation just like other modes of communication (facial expression, gesture, visual, etc.). The paper has successfully assigned psychological interpretations of all the important IPA sounds, and explained how they can be used to clarify the psychological root of any word. Semantic representations are allotted by nature; hence the author has kept his view observational. Why nature has given velar sounds for ‘clarifying’ a perception, is a question like why nature has given gravitation to a mass, which cannot be answered. The theory can be tested by applying the semantic representations of the phonemes used in different words, and finding if the pragmatic meanings of the words are explained or not. The paper presents 245 words for which the result has been found satisfactory.

## Electronic supplementary material

Below is the link to the electronic supplementary material.

Supplementary material 1 (DOCX 108 kb)
