# Supplementary material for: Psychological Model of Phonosemantics
Source: J Psycholinguist Res. 2020 Apr 22;49(3):453–74. doi: 10.1007/s10936-020-09701-y (PMC7253384; doi:10.1007/s10936-020-09701-y)
Supplement: Supplementary file 1 — Supplementary material 1 (DOCX 108 kb) [file 10936_2020_9701_MOESM1_ESM.docx]

**Appendix - A**

**List of phonesthemes** / fl /- (Please follow the rules Fig 4)

**example** /IPA/ [dictionary meaning] – psychological representation; *pragmatic meaning after making the arbitrary selection from the possible meanings in the psychological representation.*

**flag** /flæ g/ [freely waving] – /g/ clarity /flæ/ visibility of free expanding motion; *clarity of visibility of waving (free expanding motion).*

**flail** /fleɪl/ [a length moving feely] – /l/ length in /eɪ/ visible indication of /fl/ unrestricted motion; *length in visible indication of free (unrestricted) motion*; *a long object having free motion*.

**flame** /fl eɪ m/ **-** [burning gas or vapor] – /m/ submitted availability of the /eɪ/ visible indication of /fl/ unrestricted expansion; *submitted availability of the visible indication of fire (unrestricted expansion).*

**flap** /flæ p/ [hinge that allows unrestricted motion] – /p/ approving the /æ/ visibility of /fl/ unrestricted motion; *approving the visibility of unrestricted motion*.

**flare** /fl ɛə r/- [sudden burst of flame] – /r/ involvement of /ɛə/ visibly available flame (existence) in /fl/ unrestricted expanded availability; *involvement of visibly available flame in unrestricted spread (expanded availability).*

**flash** /flæʃ/ [sudden burst of bright light] – /ʃ/ physical lively expression /æ/ visibility of /fl/ unrestricted emission; *energetic (physical lively expression) visibility of sudden (unrestricted) emission.*

**flat** /flæ t/ [place or surface in a level] – /t/ occupation in /flæ/ visibility of a free available expansion; *occupation in visibility of a level (free available expansion).*

**flee** /fl i/ [to run away] – /fli/ exposing unrestricted motion; *exposing fast (unrestricted) motion;*

**flex** /flɛks/ [to bend a long object; flex] – /ks/ conscious expression of /ɛ/ visibly available /fl/ unrestricted expanded availability; *conscious expression of visibly available flexible (unrestricted) length (expanded availability); expressing the property of flexible length.*

**flick** /flɪk/ [rapid motion; a sudden light] – /k/ consciousness in /flɪ/ visible sudden motion/glow; *awareness (consciousness) in visible sudden motion / glow.*

**flicker** /flɪkər/ [an unsteady flame or light; unsteady movement] – /r/ involvement in /flɪkə/; *involvement in* /flɪkə/.

**flinch** /flɪnʧ/ [the act of alertly drawing back suddenly] – /n→ɲʧ/ alert liveliness in the /flɪ/ visibly unrestricted emotion; *alert liveliness in the visibly unrestricted emotion*.

**fling** /flɪ ŋ / [to move violently; fling] – /ŋ/ full of liveliness (strength; pleasure) in /flɪ/ visibly free motion; *full of liveliness (strength*; *pleasure) in visibly free motion*.

**flip** /flɪp/ [to move suddenly] – /p/ acquiring /flɪ/ visibly uncontrolled motion; *acquiring visibly sudden (uncontrolled) motion.*

**flit** /flɪt/ [to move swiftly] – /t/ to flow with /flɪ/ visibly unrestricted motion; *to flow with visibly unrestricted motion*;

**flock** /flɒk/ [a large group of things] – /k/ consciousness in /ɒ/ acceptability of /fl/ free expanded availability; *consciousness in acceptability of group (free expanded availability).*

**flood** /flʌ d/ - [a great flow of water; flood] – /d/ already having /flʌ/ evolved unchecked expansion; *already having evolved unchecked expansion [of water or of anything]*.

**floor** /flɔ r/ [ground of room] –/r/ involvement in the /ɔ/ acceptable availability of /fl/ free expansion; *involvement in the acceptable availability of free expansion*.

**f****lop** /flɒp/ [word origin c. 1600, ‘fall or drop heavily’] –/p/ approval of /ɒ/ acceptability of /fl/ unrestricted motion; *approval of acceptability of fall (unrestricted motion).*

**flounce** /flaʊns/ [to go with increased motion] –/ns/ active expression of /aʊ/ acceptable entity of /fl/ unrestricted motion; *active expression of acceptable entity of unrestricted motion.*

**flourish** /fl ɜrɪ ʃ/ [to be successful] /ʃ/ believable expression /ɜrɪ/ by the visible availability of /fl/ unrestricted expansion; *believable expression by the visible availability of success (unrestricted expansion).*

**flout** /fl aʊ t/ [contemptuous remark or act] – /t/ outflow of /flaʊ/ accepted unchecked expanded availability; *speak (outflow) of accepted mistakes (unchecked expanded availability).*

**flow** /fl oʊ/ [to proceed continuously and smoothly] – /oʊ/ in the acceptable direction of /fl/ unrestricted motion; *in the acceptable direction of unrestricted motion*.

**flu** /fl u/ [fever] – /flu/ accepting inside the unchecked expansion of existent; *accepting inside the unchecked expansion of temperature (existent).*

**flurry** /flɜri/ [sudden excitement] – /i/ outflow /rɜ/ of the concentrated availability of /fl/ unrestricted emotion; *outflow of the concentrated availability of uncontrolled (unrestricted) emotion.*

**flutter** /flʌ tə r/ [to flap the wings rapidly] – /r/ involvement in /tə/ activation of /flʌ/ evolved unrestricted motion; *involvement in activation of evolved unrestricted motion*.

**fly** /flaɪ/ [movement through the air] – /flɪa/ visibly free motion entity; *visibly free mobility (motion entity).*

The above examples suggest that the psychological perception of the phonestheme / fl / can be used for pragmatic representations like *free expanding motion; unrestricted expansion; unrestricted spread; sudden illumination; free available expansion; fast motion; flexible length; sudden motion / glow; unrestricted emotion*; *free motion*; *unchecked expansion*; *unrestricted expansion; uncontrolled emotion.* The words have two common parameters of /f/ and */* l */*, where / f / is used as an adjective and / l / is used as a noun.

**Appendix - B**

**List of phonesthemes** /pr/ (Please follow the rules Fig 4)

**practice** /præ ktɪ s/ [repeated performance] – /s/ physical expression of /ktɪ/ visible conscious activation by /præ/ visibility of approvable involvement; *physical expression of learning (visible conscious activation) by practice (visibility of approvable involvement).*

**praise** /pr eɪ z/ [expression of admiration] – /z/ lively expression /eɪ/ about /pr/ approvable involvement; *lively expression about admiration (approvable involvement).*

**prance** /prɑ ns/ [springing physical motion] – /ns/ active physical expression /prɑ/ by approvable involvement; *active physical expression by approvable involvement [for a child]*

**pray** /pr eɪ/ [worship; origin - late 13c. ‘ask earnestly, beg, pray to God or saint’] – /eɪ/ visible indication of /pr/ approvable concentration; *visible indication of devotion (approvable concentration).*

**prayer** /pr ɛə r/ [act of praying to God/gods] – /r/ involvement in /ɛə/ visible availability of /pr/ approvable concentration; *involvement in visible availability of devotion (approvable concentration).*

**preach** /priʧ/ [to give earnest advice, as on religious subjects] – /ʧ/ liveliness in /i/ exposition of /pr/ approvable involvement; *liveliness in exposition of devotion (approvable involvement).*

**precis** /preɪsi/ [expression of essence; cut short] – /si/ exposing expression /eɪ/ about /pr/ approvable concentration; *exposing expression about conclusion (approvable concentration).*

**pretty** /prɪ ti/ [pleasing to eye] – /ti/ exposing the presence of /ɪ/ visibly /pr/ approvable involvement; *exposing the presence of a visibly pleasing (approvable involvement).*

**price** /praɪ s/ [consideration for purchase] – /s/ expression of /prɪ/ visibly approvable involvement for /a/ entity; *expression of consideration (visibly approvable involvement) for an entity.*

**prick** /prɪk/ - [pain due to pricking; pinch] – /k/ consciousness in /ɪ/ visible /pr/ acquirable involvement; *consciousness in visibly pervious (acquirable) involvement.*

**pride** /pra ɪ d/ [expressible history of importance] –/d/ history of /ɪ/ visible /pra/ existence of approvable involvement; *history of visible existence of admiration (approvable involvement).*

**priest** /pri st/ [activator of prayer] – /st/ expressible activation of /pri/ exposing approvable involvement; *expressible activation of exposing devotion (approvable involvement).*

**prim** /prɪm/ [origin 1680 - a formal and precise demeanor] - /m/ submissive availability of /prɪ/ noticeable approvable involvement; *demeanor (availability) of preciseness (noticeable approvable involvement).*

**prince** /prɪ ns/ [expression of sovereignty] /ns/ active expression of /prɪ/ visible approvable involvement; *active expression of visible authority (approvable involvement).*

**prism** /prɪ zə m/ [converting light into beauty] - /m/ submitted availability of /zə/ lively expression of /prɪ/ visibly approvable involvement; *light (submitted availability) with diversity (lively expression) of beauty (approvable involvement).*

**probe** /pr o ʊb/ [examine thoroughly] – /ʊb/ conditions in acceptance /o/ towards /pr/ approvable involvement; *check (conditions in acceptance) for approvable involvement.*

**profess** /prəfɛs/ [false claim of knowledge] /s/ expression for /fɛ/ visible availability of unchecked approval regarding /prə/ approvable involved existence; *expression of notion (visible availability of unchecked approval) regarding knowledge (approvable involved existence).*

**prone** /pr oʊ n/ [o.1400. c naturally inclined to] /n/ eagerness /oʊ/ towards acceptance for /pr/ approved involvement; *eagerness towards acceptance for approving (approved involvement).*

**prop** /pr ɒ p/ [origin – 15c ‘support’; providing support] – /p/ support for /ɒ/ acceptability of /pr/ security involvement; *support for acceptability of protection.*

**prove** /pr u v/ [to establish the reality/truth] – /v/ invisible sensation for /u/ acceptance of /pr/ approvable involvement; *invisible sensation for acceptance of logic (approvable involvement).*

Most of the examples suggest that the psychological perception of / pr / is “approvable involvement / concentration”. The feeling can be interpreted in a number of different arbitrary purposes like *encouragement; conclusion; pleasing; pervious involvement; admiration; devotion; authority; beauty; approvable involvement; knowledge; approving; acceptability of protection; logic.* These words seem to be different, but they all have two common parameters of /p/ and /r/.

**Appendix - C**

**List of phonesthemes** / gl / (Please follow Fig 4)

**glamour** /glæ mə r/ [origin 1720 – magic, enchantment] – /r/ involvement in /mə/ submitted availability of /glæ/ visibility of clear appearance; *involvement in submitted availability of beauty (visibility of clear appearance).*

**glare** /gl ɛə r/ [origin – shine brightly] – /r/ involvement in /ɛə/ visibly available existence of /gl/ bright emitted availability; *involvement in visibly available existence of illumination (bright emitted availability).*

**glass** /glæs/ [a transparent object] - /s/ expression of /glæ/ visibility of clear availability of [light]; *expression of transparency (visibility of clear availability of [light]).*

**gleam** / gli m / [brilliant light; brightness] – /m/ submitted availability of /gli/ exposed bright emission; *quantity (submitted availability) of exposed illumination (bright emission).*

**glean** / gli n / [to collect left out grains] – /n/ to acquire the /gli/ exposed clear appearance; *to acquire the exposed substance (clear appearance).*

**glen** /glɛn/ [a small, narrow, secluded valley] – /n/ narrowness (emptiness) in /glɛ/ visibly available clear expanded availability; *narrowness in visibly available long passage (clear expanded availability).*

**glimmer** /glɪmər/ [a faint or unsteady light] – /r/ involvement in /mə/ unapproved submission /ɪ/ visible /gl/ bright emitted availability; *involvement in unsteady (unapproved submission) visible illumination (bright emitted availability)*

**glisten** /glɪsən/ [to reflect a sparkling light] – /n/ act for /sə/ expression of /ɪ/ visible /gl/ bright emitted availability; *act for expression of visible illumination (bright emitted availability).*

**glitter** /glɪtər/ [bright; shining] – /r/ involvement in /tə/ activation of /glɪ/ visible bright emitted availability; *involvement in activation of visible illumination (bright emitted availability).*

**glob** /glɒb/ [a drop or globule of a liquid; a rounded quantity of something] – /b/ bonded in /glɒ/ the acceptability of the clear expansion; *bonded in the acceptability of the sphere (clear expansion).*

**glow** /gl oʊ/ [light emitted heated to luminosity] - /oʊ/ in the acceptable direction of /gl / clear expandable appearance; *in the acceptable direction of luminosity (clear expandable appearance).*

**glue** /glu/ [adhesive] –/u/ accepting /gl/ the clear expanded availability; *accepting the surface area (clear expanded availability).*

The examples suggest that most of the cases the psychological perception of / gl / is “clear expanded appearance” or “bright emitted availability”, which is interpreted in different arbitrary purposes like *beauty; illumination; transparency; exposed substance; long passage; sphere; luminosity; surface area.* These words seem to be different, but they all have two common parameters of / g / and / l /.

**Appendix - D**

**List of English words** (Please follow the rules Fig 4)

**advice** /æ dva ɪ s/ [an opinion offered] – /s/ expression of /dvaɪ/ visible past belief in /æ/ visibility; *expression of opinion (visible past belief) in visibility.*

**agree** /ə gri/ [to come to one opinion] – /gri/ exposing clarified involvement /ə/ by existence; *exposing affirmation (clarified involvement) by existence.*

**anger** /æ ŋgə r/ [strong feelings/emotion aroused by injustice, and wrong] – /r/ involvement in /ŋgə/ emotionally strong clarity /æ/ visibility; *involvement in impulsive (emotionally strong clarity) visibility.*

**balloon** /bə lu n/ [a bag of thin rubber inflated with air] – /n/ act for achieving /lu/ the accepted expansion of /bə/ confined/restricted existence; *act for achieving the accepted expansion of air (confined existence).*

**ban** /bæ n/ [to prohibit, forbid, or bar] – /n/ act for /bæ/ visibility of restriction; *act for visibility of restriction*.

**bar** /bɑ r/ [prohibited; origin - late 12c. "stake or rod of iron used to fasten a door or gate", 1590s. barrier over which drinks were served] – /r/ involvement in /bɑ/ restricted entity; *involvement in restricted entity*.

**barrier** /bæ riə r/ [anything built to bar passage] – /r/ involvement in /riə/ existence of exposed involvement of /bæ/ visibility of bond; *involvement in implementation (existence of exposed involvement) of prohibition (visibility of bond).*

**bat** /bæ t/ [the wooden club used in certain games to strike the ball] – /t/ activation by /bæ/ visibility of bond; *activation by hardness (visibility of bond).*

**black** /blæ k/ [absolute darkness] – /k/ clarifying the /blæ/ visibility of restricted emission; *clarifying the non-emission (visibility of restricted emission).*

**blast** /blɑ st/ [explosion] – /st/ expressing activation of /bla/ bound expandable entity; *expressing the activation of Gun powder (bound expandable* *entity)*.

**bottle** /bɒ tl/ [a portable container for holding things] – /tl/ expandable occupation for /bɒ/ acceptability of acquired existent; *expandable occupation for acceptability of acquired existent.*

**call** /kɔ l/ [to speak; to convey] – /l/ expanded emission [of sound] of /kɔ/ acceptable availability of consciousness; *expanded emission [of sound] of message (acceptable availability of consciousness).*

**catch** /kæ ʧ/ [to seize or capture] – /ʧ/ attaining the energetic existent by /kæ/ visibility of consciousness; *attaining the energetic object (existent) by visibility of alertness (consciousness).*

**chief** /ʧi f/ [the boss] – /f/ unconditional approval for /ʧɪ/ visible liveliness; *unconditional approval for authority (visible liveliness).*

**clap** /klæ p/ [to clap hands] – /p/ approval for the /klæ/ visibility of conscious expansion; *approval for the visibility of attention (conscious expansion).*

**confuse** /kə nfy u z/ [fail to distinguish; to make unclear] – /z/ lively expression of /u/ acceptance of /nfy/ active unchecked view in /kə/ consciousness; *lively expression of acceptance of uncertain (active unchecked) view in consciousness*.

**czar** /zɑ r/ [an emperor] – /r/ involvement of /zɑ/ lively expressing entity; *involvement of lively expressing entity*.

**dam** /dæ m/ [obstruct the flow of water] – /m/ submitted availability of /dæ/ visibility of stopped flow; *submitted availability of visibility of stopped flow*.

**dew** /dyu/ [moisture condensed from the atmosphere; dew] – /dyu/ accepting the affirmative end; *accepting the condensation (affirmative end).*

**diary** /dɛ ə ri/ [book for daily record of events] – /ri/ exposable involvement of the /dɛə / visibly available past; *writing (exposable involvement) of the visibly available past.*

**dive** /daɪ v/ [to plunge into water; especially headfirst] – /v/ hiding the existence /daɪ/ by visible activation; *hiding the existence by visible activation*.

**enjoy** /ɛ nʤɔ ɪ/ [to feel joy; getting energy] – /ɪ/ manifestation of /nʤɔ/ acceptable available active aliveness in /ɛ/ visible availability; *manifestation of pleasure (acceptable available active aliveness) in visible availability.*

**fact** /fæ kt/ [something that actually exists] - /kt/ conscious occupation /fæ/ visibility of unconditional approval; *conscious occupation of believing (visibility of unconditional approval).*

**fast** /fɑ st/ [rapid; able to move, operate, or function quickly] – /st/ expressible flow by /fɑ/ the unchecked entity; *expressible flow by the unchecked entity*.

**flag** /flæ g/ [freely waving] – /g/ clarity of /flæ/ visibility of free available expansion; *clarity of visibility of waving (free available expansion).*

**follow** /fɒ lǝ ʊ/ [to accept as a guide or leader; believing in as a model] – /ʊ/ accepting /lǝ/ expansion of /fɒ/ acceptability of unconditional approval; *accepting expansion of faith (acceptability of unconditional approval).*

**free** /fr i/ [not under compulsion; seamless] - /i/ exposition /fr/ free involvement; *exposing free involvement.*

**gown** /ga ʊ n/ [woman`s full-length dress] – /n/ act for /ʊ/ hiding the /ga/ clear entity; *act for hiding the nude body (clear entity).*

**group** /gru p/ [number of persons considered together for the same purpose] – /p/ approval of the /gru/ accepting clear involvement; *approval of the alliance (accepting clear involvement).*

**hall** /hɔ l/ [a large room for public gatherings] – /l/ expanded /ɔ/ acceptable availability of /h/ physical space; *expanded acceptable availability of physical space*.

**have** /hæ v/ [to possess; own] – /v/ keeping of /hæ/ visibility of physical availability; *keeping of something (visibility of physical availability).*

**hill** /hɪ l/ [a natural elevation of the earth surface] – /l/ expanded availability /hɪ/ of visibly physical existence; *expanded availability of visibly physical existence; /h/ denotes physical material.*

**honour** /ɒ nr/ [high respect; honesty; fairness] – /nr/ acquired involvement in /ɒ/ acceptability of existence; *acquired involvement in acceptability of existence.*

**hug** /h ʌ g/ [to take in arms] – /g/ clarity of /hʌ/ disclosure of physical place; *clarity of disclosure of providing bodily (physical) place.*

**ignore** /ɪ gnɔ r/ [to refrain from noticing or recognizing; aside] – /r/ involvement in /gnɔ/ clear negation of acceptability of the /ɪ/ view; *involvement in neglecting (clear negation of acceptability) of the visible existence.*

**ink** /ɪ ŋk/ [a fluid for writing] – /ŋk/ lively consciousness in /ɪ/ visibility; *lively consciousness in visibility*.

**jack** /ʤæ k/ [tool for lifting car] – /k/ clarifying the /ʤæ/ visibility of power; *clarifying the visibility of power.*

**jail** /ʤ eɪ l/ [a strong place for confinement of a person; a cage] – /l/ expanded availability of /eɪ/ visible indication of /ʤ/ strong place; *expanded availability of visibly strong place*.

**jaw** /ʤɔ/ [either of two bones] – /ɔ/ acceptable availability with /ʤ/ strength; *acceptable availability with strength.*

**keep** /ki p/ [origin- before 1000 ‘to watch continuously] – /p/ approval /ki/ exposed consciousness; *approval for exposed vigilance (consciousness); keeping an eye continuously*.

**knob** /nɒ b/ [rounded handle of a door] – /b/ bond of /nɒ/ acceptability of acquisition; *bond of acceptability of acquisition*.

**lace** /l eɪ s/ [a cord for holding anything] – /s/ expression /eɪ/ about /l/ expanded availability; *expression about expanded availability*.

**lamp** /læ mp/ [a device to produce artificial light] – /mp/ submitted approval of /læ/ visibility of light; *submitted approval of visibility of light*.

**land** /læ nd/ [an area of ground] – /nd→ɳd/ spatial occupation /læ/ in visibility of available expansion; *spatial occupation in visibility of available expansion.*

**line** /la ɪ n/ [a mark long in proportion to its breadth] – /n/ emptiness in /ɪ/ visibility with /la/ expanded entity; *fine (emptiness) in visibility with long (expanded) entity.*

**no** /n oʊ/ [denial] – /oʊ/ acceptable direction of /n/ negation; *acceptable direction of negation*.

**now** /na ʊ/ [at this moment] – /ʊ/ acceptance for /na/ the action; *acceptance for the action*.

**oath** / oʊ θ/ [solemn statement; vow] – /θ/ established submission /o/ in the direction of /ʊ/ acceptance; *established submission in the direction of acceptance*.

**pass** /pɑ s/ [a permission to pass, go, or come] – /s/ expression by /pɑ/ approver; *expression made by authority*.

**push** /pʊ ʃ/ [early 1400 c., from Old French poulser, from Latin pulsare "to beat, strike, push,"; to press upon against with force] – /ʃ/ application of physical strength with /pʊ/ approvable towards inside; *application of physical strength with approvable towards inside*.

**rally** /ræ li/ [large gathering for common purpose] – /li/ exposed expansion of /ræ/ visibility of involvement; *exposed expansion of gathering (visibility of involvement).*

**see** /s i/ [look at] – /i/ exposing /s/ the expression; *exposing the expression*.

**star** /stɑ r/ [visible in the clear night sky] – /r/ involvement in /stɑ/ expressible activeness; *involvement in expressible activeness*; *the 'star' includes: player (expressible play), artist (expressible drama), sky star (expressible light)*.

**what** /wʌ t/ [asking for information; what] – /t/ activation for /wʌ/ evolved invisible expression; *activation for the evolved unknown (invisible expression).*

**wind** /w ɪ nd/ /nd→ɳd/ [air blow in natural motion] – /ɳd/ spacious flow of /ɪ/ noticeable /w/ invisible expression; *spacious flow of noticeable air (invisible expression).*

**write** /r a ɪ t/ [to form letters etc. in visible format] – /t/ activation in /aɪ/ visible entity with /r/ concentration; *activation in script (visible entity) with concentration.*

**Appendix - E**

**List of French words** (Please follow Fig 4) We have common sound for /**t**/, /t̪ʰ/, /t̪/, and /θ/.

**ami** /a mi/ [friend] - /mi/ exposing towards non-ego /a/ by the entity; *exposing respect (towards non-ego) by the entity.*

**amour** /a mu ʀ/ [love] - /ʀ/ involvement in /mu/ acceptable towards non-ego /a/ by the entity; *involvement in acceptable surrender (towards non-ego) by the entity.*

**avion** /a vj ɔ̃/ - [aeroplane] - /ɔ̃/ continuous acceptable availability of /vj/ observable hidden existence /a/ by the entity; *continuous acceptable availability of air (observable hidden existence) by the entity.*

**ballon** /ba l ɔ̃/ [balloon] /ɔ̃/ desired acceptable availability of /l/ expansion in /ba/ bound entity; *desired acceptable availability of expansion [by air] in bound entity*; *entity can be expanded as desired*.

**beau** /bo/ [beautiful] - /bo/ in the direction of acquisition [of beauty]; *direction of acquisition of beauty*.

**besoin** /bə zwɛ̃/ [need] - /zwɛ̃/ eagerness in visibly available lively expressed invisible expression /bə/ of acquisition; *eagerness in visibly available emotion (lively expressed invisible expression) of acquisition.*

**bête** /bɛ t/ [animal] - /t/ activation according to /bɛ/ visibly available confined thoughts (bond); *activation according to mental limitations*.

**binocle** /bi nɔ kl/ [telescope] - /kl/ conscious expansion in /nɔ/ acceptable available act of /bi/ exposed acquisition; *conscious expansion in the act of viewing (exposed acquisition).*

**blanc** /bl ɑ̃/ [white; no colour] - /ɑ̃/ emptiness in /bl/ acquirable available appearance; *emptiness in acquirable available appearance*; *no colour in appearance*.

**blouse** /bluz/ [blouse] - /z/ lively expression of /blu/ acceptable bound expanded appearance; *lively expression of acceptable bound breasts (expanded appearance).*

**ça** /sa/ [that; this] - /sa/ expressible entity; *expressible entity*;

**cahier** /ka je/ [ note book] - /je/ indicating the observable existent for /ka/ conscious act; *indicating the observable existent for noting (conscious act).*

**caisse** /kɛ s/ [case] - /s/ expression of /kɛ/ visibly available consciousness; *expression of discussion (visibly available consciousness).*

**ce** /sə/ [this; that] - /sə/ expressed existence; *expressed existence*.

**chaud** /ʃo/ [heat; warmth] - /ʃo/ in the direction of physically energetic experience; *in the direction of physically hot (energetic) experience.*

**chaussée** /ʃo se/ [road, pavement] - /se/ indicated expression /ʃo/ towards application of physical strength; *indicated expression towards strengthen (application of physical strength).*

**cher** /ʃɛ ʀ/ [dear; pet] - /ʀ/ involvement in /ʃɛ/ visibly available believable experience; *involvement in secured (visibly available believable) experience*.

**chez** /ʃe/ [at house] - /ʃe/ indicated place for physical aliveness; *indicated place for physical aliveness*

**chute** /ʃyt/ [fall; drop; loss] /t/ activation due to /ʃy/ affirmative application of physical aliveness; *activation due to gravitation (affirmative application of physical aliveness).*

**ciel** /sjɛ l/ [sky] - /l/ expanded availability of /sjɛ/ visibly available expressible observable existent; *expanded availability of visibly available openness (expressible observable existent).*

**clair** /klɛ ʀ/ [light] - /ʀ/ involvement in /klɛ/ visibly available conscious emission; *involvement in visibly available light (conscious emission)*.

**coût** /ku/ [price] - /k/ consciousness in /u/ accepting inside; *consciousness in purchasing (accepting inside).*

**de** /də/ [of] – /də/ occupied in existence; *related to . . .*.

**défense** /de fɑ̃ s/ [defence] - /s/ expression of /fɑ̃/ desired unconditional protection in /de/ indicated tendency; *expression of tendency of security (desired unconditional protection).*

**délicat** /de li ka/ [delicate] - /ka/ conscious entity in /li/ exposed appearance of /de/ indicating death; *alertness (conscious entity) in the exposed appearance of indicating end*.

**démon** /de m ɔ̃/ [demon] - /mɔ̃/ desired acceptable availability submission of /de/ indicating death; *perceived (desired acceptable availability) submission of spoiling (indicating death).*

**départ** /de pa ʀ/ [departure]-/ʀ/ involvement in /pa/ approvability towards /de/ indicating end; *involvement in approvability towards indicating end*

**désert** /de zɛ ʀ/ [desert] - /ʀ/ involvement in /zɛ/ visibly available lively expression of /de/ indicated end; *involvement in life (visibly available lively expression) of indicated end.*

**diable** /djɑ bl/ [devil] - /bl/ bound expanded appearance for /djɑ/ death observable entity; *bound expanded appearance for fear (death observable entity).*

**douce** /du s/ [sweet] - /s/ expression of /du/ acceptable state; *expression of acceptable state*.

**doute** /du t/ [t → t̪] [doubt] - /t̪/ submission of /du/ hidden state; *submission of hidden state*;

**enveloppe** /ɑ̃v(ə)lɔp/ [envelop] -/p/ approval of /lɔ/ acceptable availability of expanded availability for /v(ə)/ keeping inside /ɑ̃/ the desired entity; *approval of space (acceptable expansion availability) for keeping inside the desired entity.*

**est** /ɛ/ [east] - /ɛ/ visible availability; *visible availability*; *visible availability starts from the east.*

**faute** /fo t/ [fault] - /t/ activation /fo/ towards unprotected approval; *activation towards mistaken (unprotected) approval.*

**feu** /fø/ [fire] - /fø/ direct indication of free acquisition; *direct indication of free acquisition*; *fire acquires everything*.

**fille** /fi j/ [girl] - /j/ observable existence of /fi/ exposing free approval; *observable existence of beauty (exposure with free approval).*

**fleur** /flœ ʀ/ [flower] - /ʀ/ involvement in /flœ/ affirmative freelance appreciated appearance; *involvement in beauty (affirmative freelance appreciated appearance)*.

**frais** /fʀɛ/ [fresh; cool] - /fʀɛ/ visibly available free approvable involvement; *visibly available freshness (free approvable involvement).*

**gagner** /ɡa ɲe/ [to win] - /ɲe/ fullness of indicated consciousness for /ɡa/ the result; fullness of indicated consciousness for the result.

**glace** /ɡla s/ [mirror; window] - /s/ expression of /ɡla/ transparent (clear appearance) entity; *expression of transparent entity*.

**hibou** /i bu/ (h' is silent) [owl] - /bu/ accepted restriction for /i/ exposed (physical) existence. *blindness (accepted restriction) for exposed physical existence*

**honneur** /ɔn œ ʀ/ [honour] - /ʀ/ involvement in /œ/ affirmative /nɔ/ desired acceptable availability; *involvement in affirmative desired respect (acceptable availability).*

**jeune** /ʒ œ n/ [young] - /n/ acquisition of /ʒœ/ affirmative energetic experience; *acquisition of affirmative energetic experience*.

**joie** /ʒ wa/ [joy; glee] - /wa/ feel (invisible expressible entity) of /ʒ/ lively experience; *feel of lively experience*.

**joli** /ʒɔ li/ [pretty] - /li/ exposed appearance of /ʒɔ/ acceptable availability of energetic experience; *exposed appearance of acceptable availability of energetic experience.*

**jouer** /ʒ we/ [to play] - /we/ indicated invisible expression of /ʒ/ energetic experience; *indicated emotion (invisible expression) of energetic experience.*

**jus** /ʒy/ [juice] /ʒy/ affirmative lively experience; *affirmative lively experience*.

**là** /la/ [there] - /la/ available appearing entity; *available appearing entity*.

**lire** /li ʀ/ [to read] /ʀ/ involvement in /li/ exposed available appearance; *involvement in exposed readable (available appearance).*

**long** /lɔ̃/ [long] - / ɔ̃ / desired acceptable availability of /l/ expansion; *desired acceptable availability of expansion*.

**mal** /ma l/ - [evil] - /l/ available appearance of /ma/ non-approvable entity; *available appearance of non-approvable entity*.

**mari** /ma ʀi/ [husband; man] - /ʀi/ exposed involvement in /ma/ surrendered entity; *exposed involvement in wife (surrendered entity).*

**nom** /n ɔ̃/ [name] - /ɔ̃/ desired acceptable availability of /n/ fullness of identity; *desired acceptable availability of fullness of identity.*

**œil** /œ j/ [eye] - /j/ observe-ability with /œ/ affirmation; *observe-ability with affirmation*.

**orage** /ɔ ʀa ʒ/ [storm] - /ʒ/ energetic experience /ʀa/ by the involved entity in /ɔ/ acceptable available existent; *energetic experience by the dust (involved entity) in air (acceptable available existent).*

**où** /u/ [where] - /u/ accepting the hidden place; *accepting the hidden place*.

**papa** /pa pa/ [daddy] - /pa/ encouragement /pa/ by the protection entity; *encouragement by the protection entity*.

**parent** /pa ʀɑ̃/ [parents] - /ʀɑ̃/ desired involvement in /pa/ protective ness; *desired involvement in the protective ness*.

**pensée** /pɑ̃ se/ [thought] -/se/ specific expression for /pɑ̃/ desired approval; *specific expression for desired approval*.

**que** /kə/ [that] - /kə/ explainable existence; *explainable existence*.

**qui** /ki/ - [who] - /ki/ exposed explanation; *exposed explanation*.

**quoi** /k wa/ [what] -/k/ conscious /wa/ invisible expressible entity; *answerable (conscious) invisible expressible entity; question mark.*

**stylo** /sti lo/ [pen] - /lo/ towards available appearance by /sti/ exposing the expressible activation; *towards readable (available appearance) by writing (exposing the expressible activation).*

**Appendix - F**

**List of German words** (Please follow Fig 4)

**Akten** /a kte n/ [documents] – /n/ acquisition of /kte/ indicated conscious activation in the /a/ entity; *acquisition of documentation (indicated conscious activation) in the entity.*

**auch** /a u ϰ/ [also; too] - /ϰ/ expressible consciousness for /u/ acceptance inside /a/ by the entity; *expressible consciousness for acceptance inside by the entity*; *need more*;

**Band** /bæ ɳd/ [volume] - /ɳd/ spatial state in /bæ/ visibility of acquisition; *spatial state in visibility of acquisition*.

**Belege** /bə leː k/ [documentary proof] - /k/ consciousness in /bə leː/ specific appearance of acquisition; *consciousness in document (specific appearance of acquisition).*

**Bischof** /bɪ ʃɔ f/ [bishop] - /f/ free approval of /ʃɔ/ acceptable availability of alive sensation of /bɪ/ ethics (visible bond); *free approval of sermon (acceptable availability of alive sensation) of ethics.*

**blutend** /bluː tn/ [bleeding] - /tn/ active flow from /bluː/ inside existing bound expansion [of blood]; *active flow from inside existing bound expansion [of blood or anything]*.

**Bruder** /br uː dɐ/ [brother] - /dɐ/ having state of /uː/ inner accepted /br/ bound involvement; *having state of inner accepted genetic (bound) involvement.*

**Buch** /buːϰ/ [book] - /ϰ/ expressible consciousness towards /buː/ inner accepted beliefs; *expressible consciousness towards knowledge (inner accepted* *beliefs)*.

**das** /da s/ [the] - /s/ expression of /da/ existing entity; *expression of existing entity*.

**dem** /deː m/ [that] - /m/ submitted avail-ability of /deː/ indicated existent; *submitted availability of indicated existent*.

**die** /diː/ [the] - /diː/ exposed existent; *exposed existent*.

**er** /eː ɐ/ [he] - /ɐ/ entity related to /eː/ specific indication; *entity related to specific indication*.

**es** /ɛs/ [it] - /s/ expression of /ɛ/ visible availability; *expression of visible availability.*

**Feuer** /fe ʊə r/ [fire] - /r/ involvement in /ʊə/ acceptance of /fe/ indication of unchecked acquisition; *involvement in acceptance of flame (indication of unchecked acquisition).*

**Fluß** /flʊ s/ [river] - /s/ expression of /flʊ/ acceptable free acquisition of expansion; *expression of acceptable free acquisition of expansion. free expansion of water*.

**Fullers** /f ʏl ɐ/ [pen] - /ɐ/ doer of /ʏl/ affirmative expanded appearance /f/ free approval [of ink]; *doer of affirmative expanded appearance with fluentness (free approval) of ink.*

**für** /fyːɐ/ [for] - /ɐ/ having /yː/ affirmation in /f/ free acquisition; *having affirmation in free acquisition.*

**gegen** /ɡeː ɡn/ [against] - /ɡn/ clear negativity /ɡeː/ towards indicated clear; *clear negativity towards subject (indicated clear).*

**Glaube** /ɡlau bə/ [belief] - /bə/ bond with /ɡlau/ inner accepted clear expanded entity; *bond with known (inner accepted clear expanded entity).*

**Gut** /ɡuː t/ [property; nature] - /t/ occupation of /ɡuː/ inner accepted clarity; *occupation of inner accepted clarity.*

**hier** /hiː ɐ/ [here] - /ɐ/ being /hiː/ exposed physical place; *being at exposed physical place*.

**house** /ha u zǝ/ [house] - /zǝ/ lively expression of /u/ acceptance inside /ha/ the physical space; *lively expression of acceptance inside the premises (physical space).*

**Hügel** /hyː ɡl/ [hill] - /ɡl/ shaped expansion of /hyː/ affirmative physical existence; *shaped expansion of affirmative physical existence*.

**Husten** /huː stn/ [cough] - /stn/ expressible activated act of /huː/ inner physical existence; *cough produces due to taking out something, which lies inside the throat*.

**ihr** /iː ɐ/ [their; her] - /ɐ/ having relation with /iː/ the exposed existence; *relation with exposed existence*.

**ist** /ɪ st/ [is] - /st/ expressible state of /ɪ/ direct existent; *expressible state of direct existent*.

**ja** /ja/ [yes] - /j/ affirmative /a/ entity; *affirmation*.

**Jäger** /jɛː ɡɐ/ [hunter; rifleman] - /ɡɐ/ having clarity of /jɛː/ visibly available observable existence; *having clarity of target (visibly available observable existence).*

**jung** /jʊ ŋ/ [young] - /ŋ/ fullness of liveliness in /jʊ/ accepting the observable existence; *fullness of liveliness in accepting the aim (observable existence).*

**kein** /kei n/ [no; not any; none] - /n/ emptiness in /kei/ exposing of the indicated consciousness; *emptiness in exposing of the indicated consciousness; everything is empty*.

**Kind** /kɪ nt/ [baby] - /nt/ empty activation in /kɪ/ visible consciousness; *minimum (empty) activation in visible consciousness; minimum consciousness.*

**Land** /la nt/ /nt→ɳt/ [land; country] - /ɳt/ spatial occupation in /la/ available expanded entity; *spatial occupation in available expanded entity*.

**Mädel** /mɛː dl/ [girl] - /dl/ existing expansion of /mɛː/ visibly submissive availability; *existing expansion of visibly submissive availability; girls are submissive in nature.*

**Maler** /maː lɐ/ [painter] - /lɐ/ doer of expanded appearance /maː/ by submitted entity; *having expanded appearance by paint (submitted entity).*

**Meister** /maɪ stə/ [master] - /stə/ expressible activation with /maɪ/ visible submissive entity; *working (expressible activation) with labours (visible submissive entity).*

**Mühle** /myːlə/ [mill] - /lə/ expanded availability for /myː/ affirmative raw submitted availability; *expanded availability for affirmative raw material (submitted availability).*

**Mutter** /mʊtɐ/ [Mother] - /tɐ/ having activation for /mʊ/ acceptable submitted availability; *having activation for feeding (acceptable submitted availability).*

**nach** /naːϰ/ [according to] - /ϰ/ expressible consciousness /naː/ in identified entity; *expressible consciousness in identified entity*.

**Name** /n eɪ m/ [name] - /m/ submitted availability /eɪ/ about /n/ identity; *submitted availability about identity*.

**nase** /naː zə/ [nose] - /zə/ lively expression of /naː/ acquisition capability; *lively expression of acquisition capability [of air]*.

**neu** /nɔ y/ [new] - /y/ affirmation in /nɔ/ acceptable availability for acquisition; *affirmation in acceptable availability for acquisition*; *now it is available*.

**Nicht** /nɪ çt/ [not] - /çt/ lively sensorial tendency in /nɪ/ visible negation; *lively sensorial tendency in visible negation*.

**nichts** /nɪçts/ [nothing] - /s/ expression of /çt/ lively sensorial tendency in /nɪ/ visible negation; *expression of lively sensorial tendency in visible negation*.

**nie** - /niː/ [never] - /niː/ continuously exposing negation; *continuously exposing negation*.

**nun** - /nu n/ [now] - /n/ act of /nu/ accepting the act; *act of starting (accepting) the act.*

**nur** /nuː ɐ/ [only] - /ɐ/ having /nuː/ emptiness inside existing; *having emptiness except inside existing.*

**ohne** - /oːnə/ [without] - /nə/ negative /oː/ towards; *towards negation*.

**Platz** /pla ts/ [place; space] - /ts/ occupiable expression of /pla/ approvable expanded entity; *occupiable expression of liveable (approvable) land (expanded entity).*

**Priester** /priː st ɐ/ [priest] - /ɐ/ doer of /st/ expressible activation of /priː/ exposed approvable involvement; *doer of expressible activation of prayer (exposed approvable involvement).*

**Religion** /re li ɡioː n/ [religion] - /n/ act /ɡioː/ in the direction of exposed clarity of /li/ exposed emotion towards the /re/ indicated involvement; *act in the direction of doctrine (exposed clarity) of sect (/li/ exposed emotion towards the /re/ indicated involvement).*

**Richter** /rɪ çt ɐ/ [judge] - /ɐ/ doer of /çt/ precise activation within /rɪ/ visible involvement; *doer of precise activation within evidence (visible involvement).*

**Schall** /ʃa l/ [sound] - /l/ expanded emission /ʃa/ by the application of physical strength; *making of the expanded availability of sound*.

**Schein** /ʃ a i n/ [certificate] - /n/ act of /i/ exposing /a/ by the /ʃ/ physical lively expression; *act of exposing by writing (physical lively expression).* [glow] - /n/ act of /i/ exposing /a/ by the /ʃ/ physical lively expression; *act of exposing by energy (physical lively expression).*

**schwanger** /ʃva ŋ ɐ/ [pregnant] - /ɐ/ having /ŋ/ fullness of aliveness in /a/ the entity of /ʃv/ hidden physical experience; *having fullness of aliveness in the entity of womb (hidden physical experience).*

**selige** /zeː lɪ ɡə/ [blessed] - /ɡə/ clarity in /lɪ/ exposed appearance of /zeː/ indicating the lively expression; *clarity in speaking (exposed appearance) of the blessing (indicating lively expression).*

**siehe** /ziː ə/ [see] - /ə/ existence of /ziː/ exposure of lively expression; *existence of exposure of lively expression*.

**so** /zoː/ [as, so, thus, such] - /zoː/ towards lively expression; *towards established (lively) expression.*

**Soldat** /zɔ ldaː t/ [soldier] - /t/ activation of /ldaː/ expanded execution entity with /zɔ/ acceptable availability of lively expression; *activation of expanded execution act with confidence (acceptable availability of lively expression).*

**Stadt** /ʃta t/ [city] - /t/ occupation by /ʃtaː/ lively expressed activation entities; *occupation by live (lively expressed activation) entities.*

**wo** /wə ʊ/ [where] - /ʊ/ acceptability of /wə/ invisible expression existence ; *acceptability of unknown place (invisible expressible existence).*

**Appendix - G**

**List of word ‘father’ in different languages** (Please follow Fig 4)

Chinese → fuxin /fʊ ʃɪ n/ - /n/ act /ʃɪ/ visible psychological strength of /fʊ/ unconditional approvable acceptance; *acquisition of visible psychological strength of encouragement (unconditional approvable acceptance).*

English → father /fɑ: ðǝ r/ - /r/ involvement in /ðǝ/ [expressed] submission /fɑ:/ unconditional approving entity; *involvement in [expressed] submission of encouragement (unconditional approving entity).*

French → père /pɛ ʀ/ - /ʀ/ involvement /pɛ/ visibly available approval with conditions; *involvement in encouragement (visibly available approval with conditions).*

German → vater /fa tǝ r/ - /r/ involvement /tǝ/ activation /fa/ by unconditional approver; *involvement in activation by courage (unconditional approver).*

Hindi → pitā /pɪ t̪ɑ/ - /t̪ɑ/ submission /pɪ/ visibly conditional courage; *submission of visibly conditional courage*.

Italian → padre /pa dre/ - /dre/ indicative submitted involvement /pa/ of conditional encouragement; *indicative submitted involvement of conditional encouragement*; providing encouragement

Japanese → chichi /ʧɪ ʧɪ/ - /ɪ/ visible /ʧ/ liveliness; *visible liveliness*.

Spanish → padre /pa dre/ - /dre/ indicative submitted involvement /pa/ by conditional encouragement; *indicative submitted involvement of conditional encouragement*; providing encouragement.

**Appendix – H**

**List of Animal Sounds** (Please follow the Fig 4, animal sounds cannot be read in sequence)

**buzz** [a bee’s sound] [Wiktionary: 2018]^[59]^ /bʌz/ – /z/ lively expression of /bʌ/ evolved protection; *efforts (lively expression) to protect the nest.*

**meow** [a cat’s sound] /miɑʊ/ [Wiktionary: 2018]^[60]^ - /ʊ/ acceptance /ɑ/ by the entity of /i/ exposed /m/ surrender; *ready to be a pet.*

**cock-a’doodle-doo** [a rooster’s sound] /kɒkǝd: dǝl du:/ [Wiktionary: 2018]^[61]^ - /u:/ accepting /d/ existing, /l/ expansion of /d:/ existing, /kǝ/ consciousness in /ɒ/ acceptability of /k/consciousness; *acceptance of expanding the existing consciousness; expanding the consciousness; awakening.*

**moo** [a cattle’s sound] **-** /mu:/ [Wiktionary: 2018]^[62]^ - /u:/ keeping inside the /m/ available substance; *keeping milk inside.*

**bhon** [an Indian [dog’s](http://en.wikipedia.org/wiki/Dog) sound] **-** /bhon/ [Wikipedia: 2018]^[63]^ - /n/ action /o/ towards /h/ physical /b/ protection; *providing protection to the territory.*

**hee-haw** [a donkey’s sound] /hi: hɔ̰̃ː/ [Wiktionary: 2018]^[64]^ - /ɔ̰̃ː/acceptability of /h/physique and /i:/exposing the /h/physique; *life moves only for the physique.*

**neigh** [a horse’s sound] /neɪ/ [Wikipedia: 2018]^[65]^ - /ɪ/ visibility of /e/ indicated /n/ eagerness; *The horse has a desire to do something.*

**a gentle neigh** [a horse’s sound] /wɪni/ [Wikipedia: 2018]^[66]^ - /i/ exposing the /n/ eagerness with /ɪ/ visibly /w/ invisible expression; *showing eagerness peacefully.*

**chatter** [a monkey’s sound] /ʧætǝ/ [Wikipedia: 2018]^[67]^ - /tǝ/ tendency of /ʧæ/ visibility of liveliness; *tendency of* *exposing energy.*

**squawk** [a parrot’s sound] /skwɔ:k/ [Wikipedia: 2018]^[68]^ - /k/ consciousness in /ɔ:/ acceptability in /w/ invisible expression of /sk/ expressible consciousness; *consciousness in memorizing message.*

**bark** [a Bengali [fox’s](http://en.wikipedia.org/wiki/Fox) sound] /huɑk:ɑ-huɑ/ - /huɑ/ territory (physical inner accepting entity) /kː/ continue consciousness (alert) for /huɑ/ territory; *continuous consciousness for the territory.*

**bark** [a Japanese [fox’s](http://en.wikipedia.org/wiki/Fox) sound)] /kon/ - /k/ consciousness /o/ towards /n/ acquiring; *consciousness towards acquiring the territory.*

**clap** [a human clapping sound] /tlp/ - /t/ activation /l/ expansion /p/ approval; *activation in expanding the approval; encouraging or appreciating the task.*

**kiss** [a human kissing sound] /pʊʧ/ **-** /pʊ/ accepting approval /ʧ/ liveliness; *accepting the approval of liveliness.*

**weeping** [a human weeping sound] /ɑ ː̃/ **-** /ː/ continue /ǝ̃/ desiring /ɑ/ entity; *continuous desire in the entity; crying.*

**whistle** [a human whistle sound] /slf / **-** /s/ physical expression /l/ available expansion /f/ unconditional approval; *unconditional approval of expanded (long distance) physical sound (expression).*

**Appendix – I**

**List of Physical Sounds** (Please follow Fig 4, physical sounds cannot be read in sequence)

**ahem** [a cough sound] /ɑhem/ - /m/ unacquirable substance /e/ indicating /h/ physical /ɑ/ form; *physical form of unacquirable substance.*

**blast** [bomb explosion] /blæst/ - /st/ expressible activation of /æ/ visibility of /bl/ bound expansion; *a bound body is expanded for activation.*

**yalpa** [drop in water] /ylp/ - /y/ affirmation /l/ available expansion /p/ approval; *affirmative approval of the expanded wave; creating a wave on water surface.*

**dap** [drop on floor] /ɽp/ - /ɽ/ activated acceleration /p/ acceptance; *after striking the floor, one part of water activates all around, and the second part of water accepts the floor; If activation is strong the sound of /ɽ/ will be strong and if the acceptance is strong, the sound of /p/ will be strong.*

**splatt** [lightning] /splɑtt/ - /tt/ acceleration of /lɑ/ expansion of /sp/ light (approval to the expression); *expanding the light.*

**yoooo** [moan of the wind] /yooo/ - /ooo/ towards /y/ affirmative; *continuous towards affirmative.*

**chiii** [slipping of belt on pulley] /ʧː/ - /ʧː/ continuous deriving energy; *continuous deriving heat.*

**slump** [water fall] /slǝmp/ - /mp/ available approval of /slǝ/ expressible expansion; *acceptance of flow in (lower) length.*

**a sound of drum in a military parade** /ŋ/ ***-*** the sound of drum / ŋ / enhances the enthusiasm (fullness of liveliness) in solders
